# Supplementary material for: Smooth Interpolating Curves with Local Control and Monotone Alternating Curvature
Source: Comput Graph Forum. 2022 Oct 6;41(5):25–38. doi: 10.1111/cgf.14600 (PMC9827861; doi:10.1111/cgf.14600)
Supplement: Supplementary file 1 — Supplement Material [file CGF-41-25-s001.zip › Local-Smooth-Interpolating-MonoCurvature/extern/clothoids/submodules/Utils/docs/rang.html]

rang - Colors for your Terminal — Utils v1.2

### Navigation

- index
- toc
- next
- previous
- Utils »
- rang - Colors for your Terminal

# rang - Colors for your Terminal¶

Note

This is an adaptation of original rang documentation
(see rang).
The code now is not header only as the original one
to avoid windows header conflict in complex projects.

**Example usage**

```
#include "rang.hpp"

using namespace std;
using namespace rang;

int main()
{
    cout << "Plain old text"
         << style::bold << "Rang styled text!!"
         << style::reset << endl;
}
```

---

*Rang* uses iostream objects - `cout`/`clog`/`cerr` to apply
attributes to output text. Since *rang* aims to support both windows and
unix like systems, it takes care of the os specific details and tries to
provide a uniform interface. Due to incompatiblities b/w different OS
versions, not all kinds of attributes are supported on every system so
rang will try to skip the ones which might produce garbage(instead of
pushing random ANSI escape codes on your streams). Detection of tty is
also handled internally so you don’t need to check if application user
might redirect output to a file.

> **Need support for non-ansi terminals? Check
> out**Termdb**which
> supports virtually all terminals and their capablities.**

Apart from setting text attributes, you can also ask rang to override
its default behaviour through these methods -

```
void rang::setControlMode(rang::control);
```

where `rang::control` takes - `control::Auto` - Automatically
detects whether terminal supports color or not(**Default**) -
`control::Off` - Turn off colors completely - `control::Force` -
Force colors even if terminal doesn’t supports them or output is
redirected to non-terminal

```
void rang::setWinTermMode(rang::winTerm);
```

where `rang::winTerm` takes - `winTerm::Auto` - Checks for newer
windows and picks Ansi otherwise falls back to Native(**Default**) -
`winTerm::Native` - This method is supported in all versions of
windows but supports less attributes - `winTerm::Ansi` - This method
is supported in newer versions of windows and supports rich variety of
attributes

Supported attributes with their compatiblity are listed below -

**Text Styles**:

| Code | Linux/Win/Others | Old Win |
| --- | --- | --- |
| `rang::style::bold` | yes | yes |
| `rang::style::dim` | yes | no |
| `rang::style::italic` | yes | no |
| `rang::style::underline` | yes | no |
| `rang::style::blink` | no | no |
| `rang::style::rblink` | no | no |
| `rang::style::reversed` | yes | yes |
| `rang::style::conceal` | maybe | yes |
| `rang::style::crossed` | yes | no |

**Text Color**:

| Code | Linux/Win/Others | Old Win |
| --- | --- | --- |
| `rang::fg::black` | yes | yes |
| `rang::fg::red` | yes | yes |
| `rang::fg::green` | yes | yes |
| `rang::fg::yellow` | yes | yes |
| `rang::fg::blue` | yes | yes |
| `rang::fg::magenta` | yes | yes |
| `rang::fg::cyan` | yes | yes |
| `rang::fg::gray` | yes | yes |

**Background Color**:

| Code | Linux/Win/Others | Old Win |
| --- | --- | --- |
| `rang::bg::black` | yes | yes |
| `rang::bg::red` | yes | yes |
| `rang::bg::green` | yes | yes |
| `rang::bg::yellow` | yes | yes |
| `rang::bg::blue` | yes | yes |
| `rang::bg::magenta` | yes | yes |
| `rang::bg::cyan` | yes | yes |
| `rang::bg::gray` | yes | yes |

**Bright Foreground Color**:

| Code | Linux/Win/Others | Old Win |
| --- | --- | --- |
| `rang::fgB::black` | yes | yes |
| `rang::fgB::red` | yes | yes |
| `rang::fgB::green` | yes | yes |
| `rang::fgB::yellow` | yes | yes |
| `rang::fgB::blue` | yes | yes |
| `rang::fgB::magenta` | yes | yes |
| `rang::fgB::cyan` | yes | yes |
| `rang::fgB::gray` | yes | yes |

**Bright Background Color**:

| Code | Linux/Win/Others | Old Win |
| --- | --- | --- |
| `rang::bgB::black` | yes | yes |
| `rang::bgB::red` | yes | yes |
| `rang::bgB::green` | yes | yes |
| `rang::bgB::yellow` | yes | yes |
| `rang::bgB::blue` | yes | yes |
| `rang::bgB::magenta` | yes | yes |
| `rang::bgB::cyan` | yes | yes |
| `rang::bgB::gray` | yes | yes |

**Reset Styles/Colors**:

| Code | Linux/Win/Others | Old Win |
| --- | --- | --- |
| `rang::style::reset` | yes | yes |
| `rang::fg::reset` | yes | yes |
| `rang::bg::reset` | yes | yes |

---

**My terminal is not detected/gets garbage output!**

Check your env variable `TERM`’s value. Then open an issue
here and make sure to
mention `TERM`’s value along with your terminal name.

**Redirecting ``cout``/``cerr``/``clog`` rdbuf?**

Rang doesn’t interfere if you try to redirect `cout`/`cerr`/`clog`
to somewhere else and leaves the decision to the library user. Make sure
you’ve read this
conversation
and check out the example code
here.

### Quick search

### Table of Contents

- rang - Colors for your Terminal
- Zstr
- C++ API

«
hide menu

menu
sidebar
»

### Navigation

- index
- toc
- next
- previous
- Utils »
- rang - Colors for your Terminal

© Copyright 2021, Enrico Bertolazzi.
Created using Sphinx 4.2.0.
